# Repetition‐dependent acute cardiopulmonary responses during intensity‐matched squats in males

**DOI:** 10.1113/EP092363

**Published:** 2025-02-22

**Authors:** Johannes Lässing, Sonja Hummelmann, Maxi Kramer, Ulrich Laufs, Sven Fikenzer, Roberto Falz

**Affiliations:** ^1^ Department of Exercise Science & Sports Medicine Martin Luther University Halle‐Wittenberg Halle (Saale) Germany; ^2^ Institute of Sports Medicine and Prevention University of Leipzig Leipzig Germany; ^3^ University Leipzig, Medizinische Fakultät Leipzig Germany; ^4^ Human‒Machine‐Interaction Magdeburg‐Stendal University of Applied Science Magdeburg Germany

**Keywords:** blood pressure response, cardiovascular response, heart rate, squat, strength training, strength–endurance continuum

## Abstract

The ‘strength–endurance continuum’ is a key concept in strength training (ST). Although cardiopulmonary responses have seldom been reported in conjunction with ST, this repeated‐measurement study examined acute blood pressure and haemodynamic responses continuously depending on the number of repetitions but without changing the intensity. Fifteen healthy male participants (21.6 (2.0) years; mean (SD)) performed an incremental exercise test and a 3‐repetition maximum test (3‐RM) on a Smith machine. They were then randomly assigned to three ST sessions involving 10, 20 and 30 repetitions at 50% of their 3‐RM. Blood pressure (vascular unloading technique) and cardiopulmonary responses (spirometry and impedance cardiography) were continuously monitored. Heart rate (121 (10) vs. 139 (22) vs. 153 (13) bpm, *P* = 0.001, respectively), cardiac output (10.4 (1.9) vs. 13.6 (3.8) vs. 14.6 (3.1) L/min, *P* = 0.001, respectively) and diastolic blood pressure (113 (8) vs. 116 (21) vs. 135 (22) mmHg, *P* = 0.001, respectively) increased in the training sessions with higher repetitions. Stroke volume, systolic blood pressure and end‐diastolic volume indicated no change in peak values between training sessions. Total peripheral resistance (13.6 (2.8) vs. 11.3 (3.6) vs. 11.2 (3.1) mmHg min/L, *P* = 0.002, respectively) was significantly lower with 20 and 30 repetitions, while oxygen uptake (${\dot{V}}_{O_{2}}$: 15.5 (1.9) vs. 20.5 (4.1) vs. 20.6 (4.4) mL/min/kg, *P* = 0.001, respectively) was significantly higher. ST of moderate intensity with an exhausting number (>20) of repetitions induces strong haemodynamic responses, especially high cardiac afterload and a compensatory heart rate acceleration, which may also create a strong stimulus for cardiopulmonary adaptation.

## Abstract (highlights)

- **What is the central question of this study?**
  What are the haemodynamic effects of different numbers of repetitions in strength training when investigated independently of intensity?
- **What is the main finding and its importance?**
  Performing an exhaustive number of repetitions (>20) in strength training elicits intense, primarily chronotropic cardiac responses, similar to isometric strength exercises.

## 1 INTRODUCTION

Strength training (ST) is crucial according to recommendations and guidelines for physical activity in both healthy individuals (World Health Organization, 2018) and in the treatment of various internal diseases (Alizaei Yousefabadi et al., 2021; Virani et al., 2023; Whelton et al., 2018), especially when combined with endurance training (Cornelis et al., 2016; Fleg et al., 2013; Kirkman et al., 2022; Williams et al., 2007).

The reactions at the cellular level, as well as organic and integral long‐term effects and chronic adaptations of the musculoskeletal and cardiovascular systems, have been thoroughly investigated (Bell et al., 2000; Chilibeck et al., 2002; Evans, 1985; Hansen et al., 2009; O'Bryan et al., 2022; Toigo & Boutellier, 2006). However, haemodynamic responses, such as stroke volume or blood pressure, have not been conclusively reported in ST. Previous studies have primarily focused on the haemodynamic reaction to endurance training (Gayda et al., 2012; Lepretre et al., 2004) or comparison between bodyweight strength exercises (e.g., squats or push‐ups) and endurance training (Falz et al., 2019). However, ST involving heavy additional weights or isometric exercises has significantly increased blood pressure (MacDougall et al., 1985; Taylor et al., 2017). This effect is especially notable when combined with breath‐holding or the Valsalva manoeuvre (Niewiadomski et al., 2012; Perry & Lucas, 2021; Perry et al., 2014). The findings suggest that resistance training significantly affects cardiovascular stress.

Furthermore, characterising acute haemodynamic response in ST is challenging due to markedly different training protocols in physiological studies, including the muscle groups activated, exercise execution (dynamic vs. isometric, bodyweight vs. free‐weight vs. machine‐based), duration and intensity (Carvalho et al., 2022; de Sousa et al., 2014; Gjovaag et al., 2016; Kambic et al., 2021; Lamotte et al., 2010, 2005; Matos‐Santos et al., 2017). Muscle mass plays a crucial role in shaping the cardiovascular response during ST, as Matos‐Santos et al. (2017) outlined. Increased intensity has also revealed a proportionally more significant rise in heart rate (HR) and blood pressure (MacDougall et al., 1985; Williams et al., 2007). Overall, most of the studies were conducted within the ‘strength–endurance continuum’ and compared high load/low repetition versus low load/high repetition ST, with varying findings regarding the cardiopulmonary response (Gjovaag et al., 2016; Kambic et al., 2021; Lamotte et al., 2005). The effects of intensity and volume are, however, seldom considered independently.

In an earlier study, we examined the haemodynamic load response during standardised squats at different exercise intensities while maintaining the same number of repetitions (Lässing et al., 2023). On the other hand, few studies have explored how the ‘strength–endurance continuum’ relates to the number of repetitions at the same intensities. Beyond gaining a deeper understanding of the long‐term cardiopulmonary adaptations to ST, it is crucial to have comprehensive knowledge about the impact of individual elements of intensity and duration. This would have practical implications for both healthy individuals and especially those with cardiovascular disease.

The purpose of this randomised cross‐over designed study was to continuously monitor the haemodynamic and cardiopulmonary responses during and after ST. Our study aimed to vary the number of repetitions (10, 20 and 30 reps) at the same intensity to observe their effects. In line with previous ST studies, we anticipated the greatest impact would be apparent with 30 repetitions.

## 2 METHODS

### 2.1 Ethical approval

Our study complied with the latest version of the *Declaration of Helsinki* and was approved by the Ethics Committee of the Medical Faculty of the University of Leipzig (212/23‐ek).

### 2.2 Participants

Our sample consisted of 15 healthy, experienced male subjects who regularly engage in ST (Table 1). All participants were free of cardiac, pulmonary and inflammatory diseases or orthopaedic complaints at the assessment time. They were required to demonstrate that they could execute technically and physically the ST task correctly and a written declaration of consent was obtained from each participant.

**TABLE 1:** Baseline characteristics of study participants (*n* = 15).

Anthropometric and physical parameters			
Age (years)	26.8 (3.3)		
Height (cm)	179.1 (6.9)		
Weight (kg)	81.1 (7.5)		
BMI (kg/m^2^)	25.4 (2.6)		
LBM (kg)	67.8 (6.2)		
FM (%)	16.3 (4.5)		
Physical activity (hours per week)	7.9 (3.0)		
3‐RM (kg)	108.8 (19.1)		
IET outcomes at	VT1	VT2	*P*_max_
*P* (W/kg)	1.67 (0.16)	2.64 (0.17)	3.1 (0.2)
HR (bpm)	122.9 (10.7)	160.8 (12.9)	176.34 (12.40)
SV (mL)	91.4 (23.3)	108.6 (25.7)	130.96 (28.80)
CO (L/min)	11.2 (2.7)	17.4 (3.8)	21.6 (4.5)
SBP (mmHg)	186.1 (18.1)	218.8 (20.2)	228.4 (20.5)
DBP (mmHg)	76.3 (7.6)	74.7 (7.7)	73.6 (7.8)
TPR (mmHg min/L)	11.4 (3.4)	8.2 (2.2)	6.8 (1.9)
RR (bpm)	22.9 (5.7)	32.5 (5.4)	48.0 (6.4)
*V*_T_ (L)	2.1 (0.5)	2.6 (0.5)	2.7 (0.5)
${\dot{V}}_{E}$ (L/min)	45.5 (7.0)	83.6 (13.6)	128.3 (21.3)
${\dot{V}}_{O_{2}}$ (mL/min/kg)	22.7 (2.5)	34.2 (3.6)	41.81 (3.4)
Lac (mmol/L)	1.8 (0.7)	4.9 (2.1)	9.0 (2.2)
RPE (0–10)	4.0 (0.9)	7.4 (1.0)	9.1 (0.5)

*Note*: Values are presented as the means and standard deviation. Abbreviations: 3‐RM, 3‐repetition maximum; BMI, body mass index; CO, cardiac output; DBP, diastolic blood pressure; FM, fat mass; HR, heart rate; IET, incremental exertion test; Lac, blood lactate concentration; LBM, lean body mass; *P*, power output; *P*_max_, maximum power output; RPE, rating of perceived exertion; RR, respiratory rate; SBP, systolic blood pressure; SV, stroke volume; TPR, total peripheral pressure; ${\dot{V}}_{E}$, minute ventilation; ${\dot{V}}_{O_{2}}$, oxygen uptake; *V*_T_, tidal volume; VT1, first ventilatory threshold; VT2, second ventilatory threshold (respiratory compensation point).

The sample size was determined based on the anticipated impact of varying the number of repetitions on HR. Perry et al. (2014) found that when comparing two versus six repetitions at 60% of 6‐RM per set, there was a difference of 28 (8) versus 38 (13) bpm, indicating a Cohen's effect size of *d* = 1.0. To achieve a power of 0.9 with an α of 0.05, a minimum of 13 subjects was required for our sample.

### 2.3 Study design

**FIGURE 1:**
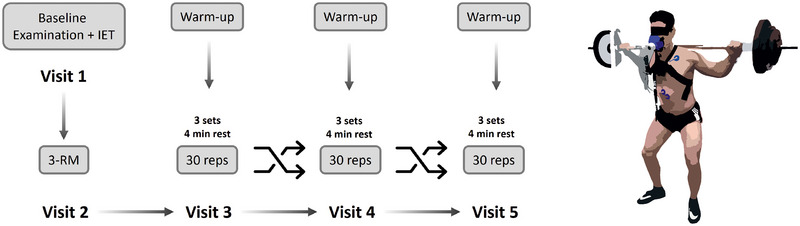
Flow chart. At visit 1, baseline examinations and the incremental exertion test (IET) were performed to ensure individual health and physical conditions. The individual 3‐repetition maximum (3‐RM) of back squat in the Smith machine was determined at visit 2. The three conditions (3 sets of 10, 20 or 30 repetitions (reps) with a resting period of 4 min between sets; intensity: 50% of 3‐RM) were performed on separate days. The order of visits 3–5 was randomised.

This study included five study visits (Figure 1). Following the initial screening (visit 1) and 3‐repetition maximum (3‐RM, visit 2) test, all participants completed three randomised training sessions involving standard squats on a Smith machine with varying numbers of repetitions (10, 20 and 30 repetitions; visits 3–5).

We assessed the clinical exclusion criteria during the preliminary examinations at visit 1 and determined maximum aerobic and strength capacity. This included taking a medical history, a lifestyle questionnaire on physical activity, smoking and alcohol consumption, echocardiography, an incremental exercise test (IET), bioelectrical impedance analysis (BIACORPUS RX 4004M, MEDI CAL HealthCare GmbH, Karlsruhe, Germany) and the 3‐RM test at visit 2.

The three ST sessions described below were then repeated in random order on different days at the same time with at least a 2‐day break to ensure sufficient recovery. Participants were also instructed not to engage in any lower body resistance training 48 h before testing and to stick to their regular diet.

#### 2.3.1 Incremental exertion test (visit 1)

All participants performed an exercise test on a semi‐recumbent ergometer (Ergoselect, ergoline GmbH, Bitz, Germany) at a standard cadence of 60–70 rpm. The IET started with a resistance of 50 W and increased by 15 W every minute until the participants reached exhaustion, which was defined by a cadence below 60 rpm, reaching a maximum rating of perceived exertion (RPE) of 10 and/or a respiratory exchange ratio (RER) above 1.1. After exhaustion, the load was reduced to 25% of the maximum power output (*P*_max_) for a 5‐min recovery. We measured the systolic (SBP) and diastolic (DBP) blood pressure (auscultatory method according to Riva‐Rocci and Korotkoff), as well as the participants’ RPE (Borg et al., 1985), oxygen saturation, and blood lactate concentration (Lac) before the test and every third minute during the test. We used electrocardiography (custo cardio BT‐A 300, custo med GmbH, Ottobrunn, Germany), spirometry (Metalyzer 3B, Cortex Biophysik GmbH, Leipzig, Germany), and thoracic impedance cardiography (PhysioFlow PF07 Q‐Link, Manatec Biomedical, Petit Ebersviller, France) to monitor the participants throughout the test continuously. The cardiopulmonary parameters' maximum values (IET 100%) represent the reference values for interpreting the subsequent repetition‐effected ST results.

#### 2.3.2 3‐Repetition maximum test (visit 2)

The 3‐RM tests followed the ACSM guidelines for 1‐RM (Liguori & American College of Sports Medicine, 2021) and used the same standardised procedure as the strength training sessions. The maximum knee flexion was limited to 90°, measured from the trochanter major to the malleolus lateralis, with a hip‐width stance. The exercise speed was standardised to a 2‐s eccentric phase, 2‐sconcentric phase, and 2‐s upright standing. There was a 4‐min resting period between sets. The load was increased in the next attempt if more than three clean repetitions were achieved. In case of an incomplete attempt (<3 repetitions), the previous load was assumed to be 3‐RM weight.

#### 2.3.3 ST (visit 3–5): 10, 20 and 30 repetitions of squats in a Smith machine

All participants started with a 10‐min warm‐up on the bicycle ergometer, followed by two sets of squats in the Smith machine without any added weight. During the subsequent training sessions, the participants used a constant weight of 50% of their 3‐RM. Each session included three sets of single‐armed back squats in the Smith machine, with three repetition numbers (10, 20 and 30). A 4‐min resting period (240 s) between sets ensured proper cardiopulmonary and metabolic recovery (de Salles et al., 2009) and allowed us to analyse the post‐exercise phase. The squats were standardised regarding knee angle (90° flexion) and movement speed. One repetition lasted 6 s. Audible and visual signals marked the three consecutive movement periods: eccentric (descending), concentric (ascending) phases and upright stance, each lasting 2 s. This results in a set duration of 60 s at 10 repetitions, 120 s at 20 repetitions and 180 s at 30 repetitions. This choice was made due to its higher measurement resolution than the usual 4‐s duration. All participants complied with at least a 48‐h exercise‐free period before the measurements.

#### 2.3.4 Measurements during ST

While exercising, the Metalyzer 3B (Cortex Biophysik) was used to record continuous data via spiroergometry, as well as PhysioFlow PF07 Q‐Link (Manatec Biomedical) for thorax impedance cardiography. HR, stroke volume (SV), cardiac output (CO), end‐diastolic volume (EDV), systolic (SBP), diastolic (DBP), and mean arterial (MAP) blood pressure were measured. We also monitored respiratory rate (RR), tidal volume (*V*_T_), minute ventilation (${\dot{V}}_{E}$), volume of oxygen uptake (${\dot{V}}_{O_{2}}$) and volume of carbon dioxide output (${\dot{V}}_{CO_{2}}$). To measure blood pressure beat to beat, we took non‐invasive measurements on the index or middle finger of the right hand with a finger cuff from Task Force Monitor (CNSystems Medizintechnik GmbH, Graz, Austria). During squats, we ensured the measurement signal's quality by fixing the hand at heart level and supporting the arm in a shoulder joint orthosis (medi arm sling, medi GmbH & Co. KG, Germany) with straps and Velcro without any muscle tension. The monitor also measured oscillometric arterial pressure on the left arm at regular intervals before the exercise and between sets to validate the measured values of arterial finger pressure. After each set, 1 min of recovery and at the end of each resting period, capillary blood samples were taken to measure blood lactate levels (Lac), and participants were asked to rate their perceived exertion (RPE). For editing purposes, we averaged the measured values of spiroergometry, impedance cardiography and blood pressure at 5‐s intervals. To evaluate the acute effect of the ST sessions, the mean value of the three sets (3 × 60/120/180 s), the mean peak value of the sets (last five repetitions, respectively 3 × 30 s), and the mean value of each first minute of the post‐exercise periods (3 × 60 s) were analysed. We calculated stroke work using the formula SW = SV × MAP (Kerkhof et al., 2018). Total peripheral resistance (TPR) was determined using the formula TPR = MAP/CO (Trammel & Sapra, 2024). The rate pressure product (RPP) was calculated using RPP = HR × SBP (Hui et al., 2000).

### 2.4 Statistical analysis

Data were analysed using Microsoft Excel 2010 and GraphPad Prism 10 (GraphPad Software, Boston, MA, USA) for Windows. All values in tables represent mean and standard deviation unless otherwise stated. Normal distribution was tested using the D'Agostino–Pearson normality test. If a normal distribution was confirmed, the statistical differences between the different repetition numbers were examined via a one‐way analysis of variance with repeated measures (RM‐ANOVA) and Tukey's *post hoc* test for multiple comparisons. In the case of non‐parametric data, we conducted the Friedman test and Dunn's *post hoc* test. A significance level of *P *< 0.05 was applied for all tests. The effect size partial eta‐squared η^2^_p_ for RM‐ANOVAs was rated according to the following benchmarks: small effect, η^2^_p_ > 0.01; medium effect, η^2^_p_ > 0.06; large effect, η^2^_p_ > 0.14 (Cohen, 1988).

## 3 RESULTS

### 3.1 Incremental exertion test

In the IET, the subjects achieved a mean (SD) maximum power output (*P*_max_) of 251.0 (29.9) W, corresponding to a relative power of 3.1 (0.2) W/kg. Table 1 presents the peak values of cardiopulmonary parameters at 100% IET and also shows values at the ventilatory thresholds during the IET.

### 3.2 Cardiopulmonary outcomes during ST sessions

The raw data for the haemodynamic parameters and the pulmonary parameters are provided as a supplement. We detected significant differences in the resting HR and DBP values before each ST session (Table 2).

**TABLE 2:** Haemodynamic and vascular response at baseline, exercise and post‐exercise period for training sessions of 10/20/30 repetitions (*n* = 15).

	**10 repetitions**	**20 repetitions**	**30 repetitions**	**Effect size η^2^_p_**	***P*‐value**
HR (bpm)					
Baseline Exercise peak Post‐exercise	85.2 (11.7) 120.6 (10.3)^b, c^ 112.8 (12.8)^b, c^	84.8 (12.2) 139.4 (21.5)^a, c^ 127.4 (22.0)^a, c^	90.1 (11.9) 152.8 (13.0)^a, b^ 141.8 (15.0)^a, b^	0.24 0.70 0.59	**0.026** **0.001** **0.001**
SV (mL)					
Baseline Exercise peak Post‐exercise	74.8 (17.8) 86.9 (16.3)^b^ 89.2 (22.8)^b^	78.6 (17.9) 96.7 (20.1)^a^ 100.2 (25.4)^a^	76.0 (17.6) 95.1 (17.2) 98.0 (21.9)	0.03 0.17 —	0.533 0.087 **0.022**
CO (L/min)					
Baseline Exercise peak Post‐exercise	6.26 (1.09) 10.44 (1.85)^b, c^ 9.98 (2.07)^b, c^	6.62 (1.39) 13.57 (3.83)^a^ 12.81 (3.89)^a^	6.74 (1.10) 14.57 (3.10)^a^ 13.95 (3.41)^a^	0.09 0.49 —	0.279 **0.001** **0.001**
EDV (mL)					
Baseline Exercise peak Post‐exercise	154.8 (29.6) 164.3 (30.6) 161.8 (30.3)	155.3 (24.9) 169.8 (28.4) 168.9 (27.9)	148.5 (28.5) 166.3 (32.9) 163.4 (28.7)	— — 0.04	0.701 0.247 0.529
SBP (mmHg)*					
Baseline Exercise peak Post‐exercise	137.6 (11.3) 173.5 (14.7) 145.3 (17.6)	146.4 (13.6) 176.6 (26.4) 138.8 (20.4)	146.4 (13.0) 184.2 (29.4) 137.5 (24.4)	0.15 0.11 0.08	0.106 0.217 0.328
DBP (mmHg)*					
Baseline Exercise peak Post‐exercise	92.9 (7.3)^c^ 113.2 (8.0)^c^ 90.5 (10.8)	96.0 (14.8) 116.4 (21.0)^c^ 84.8 (15.0)	101.6 (12.9)^a^ 134.7 (21.9)^a, b^ 94.2 (19.7)	0.22 0.44 0.18	**0.033** **0.001** **0.069**
RPP*					
Baseline Exercise peak Post‐exercise	11741 (1965)^c^ 21419 (3283)^b, c^ 16732 (2775)^c^	12473 (2489) 25154 (5416)^a, c^ 17913 (3894)	13217 (2353)^a^ 28802 (5404)^a, b^ 19614 (3772)^a^	0.24 0.69 0.34	**0.033** **0.001** **0.005**
TPR (mmHg min/L)*					
Baseline Exercise peak Post‐exercise	18.0 (3.5) 13.6 (2.8)^b, c^ 11.58 (2.37)^b, c^	17.9 (3.3) 11.3 (3.6)^a^ 9.07 (3.07)^a^	18.2 (4.4) 11.2 (3.1)^a^ 8.47 (3.01)^a^	< 0.01 0.38 0.45	0.924 **0.002** **0.0004**
SW (J)					
Baseline Exercise peak Post‐exercise	1.09 (0.25) 1.59 (0.30)^b, c^ 1.32 (0.33)	1.21 (0.32) 1.84 (0.49)^a^ 1.41 (0.40)	1.19 (0.23) 1.99 (0.52)^a^ 1.43 (0.39)	0.12 0.29 0.09	0.183 **0.013** 0.279

*Note*: Values presented as the means and standard deviation. *n = 14 (due to technical malfunction of blood pressure monitor). η^2^_p_, partial eta‐squared of the one‐way repeated measures ANOVA. *P*‐values shown in bold indicate statistical significance. ^a^Different from 10 repetitions (*P* < 0.05). ^b^Different from 20 repetitions (*P* < 0.05). ^c^Different from 30 repetitions (*P* < 0.05). Abbreviations: CO, cardiac output; DBP, diastolic blood pressure; EDV, end diastolic volume; HR, heart rate; RPP, rate pressure product; SBP, systolic blood pressure; SV, stroke volume; SW, stroke work; TPR, total periphery resistance.

**FIGURE 2:**
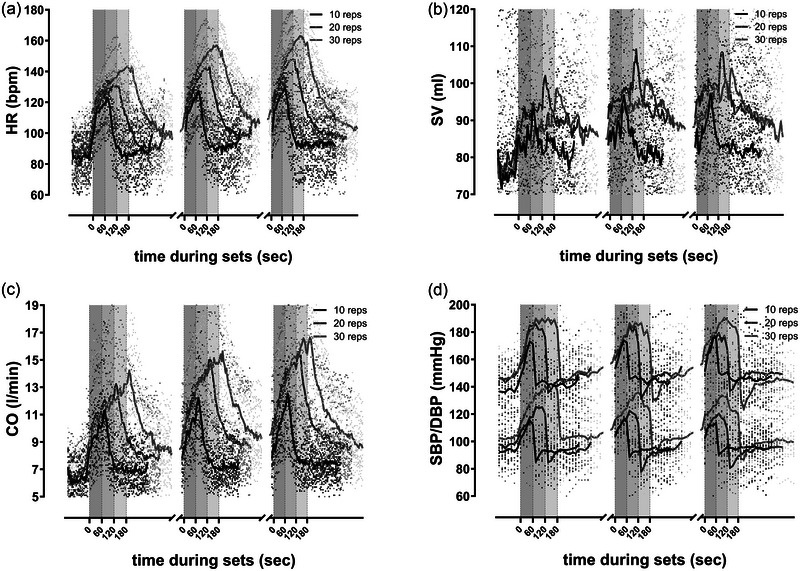
Graphs show the mean haemodynamic response during strength training sessions (10 repetitions: 214 data points during three sets/breaks in 15 participants; 20 repetitions: 250 data points during three sets/breaks in 15 participants; 30 repetitions: 286 data points during three sets/breaks in 15 participants). (a) Heart rate (HR); (b) stroke volume (SV); (c) cardiac output (CO); (d) systolic and diastolic blood pressure (SBP/DBP); *n* = 14 (due to technical malfunction of blood pressure monitor). Grey‐shaded areas indicate the exercise intervals of the strength training sessions; exercise intervals lasted 60 s for 10 repetitions, 120 s for 20 repetitions and 180 s for 30 repetitions; resting periods between all sets were 4 min. reps, repetitions.

**FIGURE 3:**
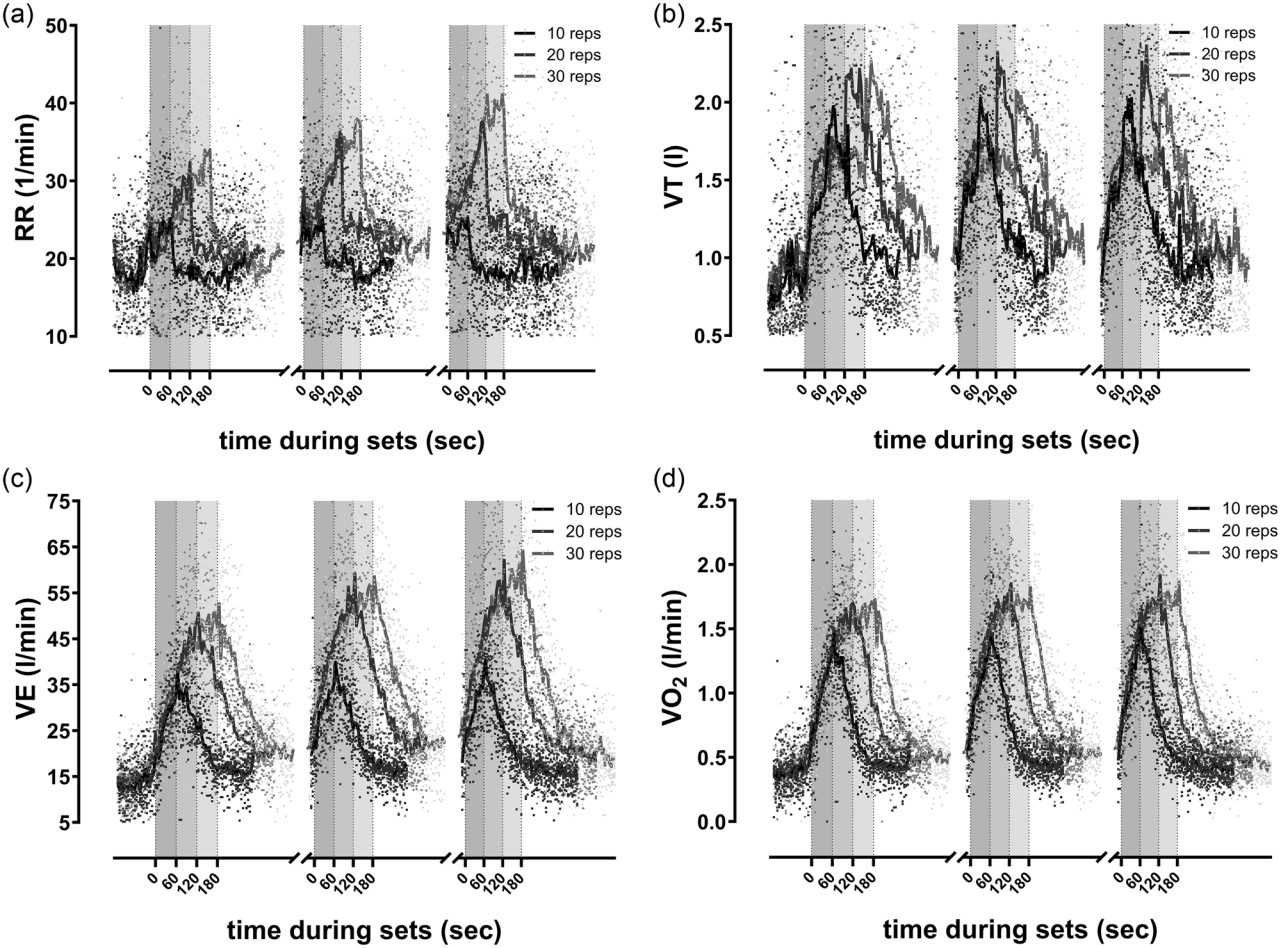
Graphs show the mean pulmonary response during strength training sessions (10 repetitions: 215 data points during three sets/breaks in 15 participants; 20 repetitions: 251 data points during three sets/breaks in 15 participants; 30 repetitions: 287 data points during three sets/breaks in 15 participants). (a) respiratory rate (RR); (b) tidal volume (*V*_T_); (c) minute ventilation (${\dot{V}}_{E}$); (d) oxygen uptake (${\dot{V}}_{O_{2}}$). Grey shaded areas indicate the exercise intervals of the strength training sessions; exercise intervals lasted 60 s for 10 repetitions, 120 s for 20 repetitions and 180 s for 30 repetitions; resting periods between all sets were 4 min. reps, repetitions.

The analysis in Table 2 shows the peak values representing the mean of the last five repetitions, respectively 30 s per set. In the third set, five participants could not complete 30 repetitions due to muscular fatigue in their back (stopping after 15–27 repetitions). Two participants failed to complete 30 repetitions in the second set, stopping after 20–22 repetitions. We included their last five repetitions (30 s) as peak values before stopping in our analysis. Figures 2 and 3 show the mean haemodynamic and pulmonary response during the training sessions.

Notably, there are significant *post hoc* differences between 20 and 30 reps in the HR (139.4 (21.5) vs. 152.8 (13.0); *P* = 0.017), DBP (116.4 (21.0) vs. 134.7 (21.9); *P* = 0.007) and MAP (141.7 (20.9) vs. 155.2 (23.6); *P* = 0.026). We also observed similar pulmonary effects at 20 and 30 repetitions, significantly increasing to 10 repetitions (Table 3). Among the pulmonary parameters, only *V*_T_ revealed lower values during the last 30 s of the sets at 30 repetitions compared to 20 repetitions (Table 3). Unlike the other pulmonary parameters, *V*_T_ does not increase throughout the sets but shows a downward trend towards the end of the sets (Figure 3). Considering the breath‐by‐breath data, note the change in the breathing pattern apparent after 9.0 (1.4) of 10 repetitions, 12.6 (2.6) of 20 reps and 18.3 (3.9) of 30 repetitions.

**TABLE 3:** Pulmonary and metabolic response at baseline, exercise and post‐exercise period for training sessions of 10/20/30 repetitions (*n* = 15).

	**10 repetitions**	**20 repetitions**	**30 repetitions**	**Effect size η^2^_p_**	***P*‐value**
RR (bpm)					
Baseline Exercise peak Post‐exercise	18.0 (4.3) 24.2 (6.1)^b, c^ 19.2 (4.1)^b, c^	18.7 (4.3) 33.2 (11.2)^a^ 23.6 (6.6)^a^	17.8 (4.1) 36.5 (10.3)^a^ 25.4 (5.5)^a^	0.05 0.54 0.50	0.464 **0.001** **0.001**
*V*_T_ (mL)					
Baseline Exercise peak Post‐exercise	0.83 (0.24)^c^ 1.44 (0.36)^b^ 1.77 (0.35)^b^	0.90 (0.27) 1.66 (0.55)^a^ 2.03 (0.58)^a^	0.93 (0.20)^a^ 1.56 (0.47) 1.94 (0.48)	— — 0.31	**0.001** **0.014** **0.007**
${\dot{V}}_{E}$ (L/min)					
Baseline Exercise peak Post‐exercise	13.46 (1.97)^b^ 32.56 (5.65)^b, c^ 31.83 (4.92)^b, c^	14.87 (2.21)^a^ 50.96 (14.45)^a^ 45.61 (13.31)^a^	15.19 (3.02) 52.95 (12.25)^a^ 47.04 (10.64)^a^	— 0.65 0.57	**0.017** **0.001** **0.001**
${\dot{V}}_{O_{2}}$ (L/min)					
Baseline Exercise peak Post‐exercise	0.39 (0.06) 1.25 (0.14)^b, c^ 1.13 (0.20)^b, c^	0.43 (0.06) 1.67 (0.39)^a^ 1.35 (0.34)^a^	0.43 (0.06) 1.68 (0.38)^a^ 1.33 (0.30)^a^	— 0.55 0.41	**0.038** **0.001** **0.001**
${\dot{V}}_{O_{2}}$ (mL/min/kg)					
Baseline Exercise peak Post‐exercise	4.78 (0.71)^b, c^ 15.5 (1.9)^b, c^ 14.0 (2.5)^b, c^	5.30 (0.63)^a^ 20.5 (4.1)^a^ 16.6 (3.7)^a^	5.33 (0.66)^a^ 20.6 (4.4)^a^ 16.3 (3.4)^a^	0.29 0.55 0.39	**0.011** **0.001** **0.001**
${\dot{V}}_{CO_{2}}$ (L/min)					
Baseline Exercise peak Post‐exercise	0.36 (0.06)^b^ 1.08 (0.14)^b, c^ 1.06 (0.18)^b, c^	0.39 (0.05)^a^ 1.60 (0.41)^a^ 1.46 (0.41)^a^	0.39 (0.05) 1.65 (0.37)^a^ 1.46 (0.33)^a^	0.23 0.66 0.60	**0.028** **0.001** **0.001**
Lac (mmol/L)					
Baseline Exercise peak Post‐exercise	0.92 (0.27) 1.37 (0.46)^b, c^ 1.81 (0.73)^b, c^	0.92 (0.27) 2.59 (1.22)^a^ 3.14 (1.38)^a^	0.96 (0.41) 2.91 (1.15)^a^ 3.43 (1.22)^a^	— 0.54 0.54	> 0.99 **0.001** **0.001**
RPE (0–10)					
Baseline Exercise peak Post‐exercise	0.00 (0.00) 3.27 (1.16)^b, c^ 2.40 (1.21)^b, c^	0.20 (0.41) 5.42 (2.03)^a, c^ 3.82 (1.47)^a, c^	0.07 (0.26) 7.09 (1.88)^a, b^ 5.36 (1.37)^a, b^	— 0.75 0.65	0.097 **0.001** **0.001**

*Note*: Values presented as the means and standard deviation. * *n* = 14 (due to technical malfunction of blood pressure monitor). η^2^_p_, partial eta‐squared of the one‐way repeated measures ANOVA. *P*‐values shown in bold indicate statistical significance. ^a^Different from 10 repetitions (*P* < 0.05); ^b^Different from 20 repetitions (*P* < 0.05). ^c^Different from 30 repetitions (*P* < 0.05). Abbreviations: Lac, blood lactate; RPE, rating of perceived exertion; RR, respiratory rate; ${\dot{V}}_{CO_{2}}$, carbon dioxide output; ${\dot{V}}_{E}$, minute ventilation; ${\dot{V}}_{O_{2}}$, oxygen uptake; *V*_T_, tidal volume.

The levels of blood lactate concentration reached a maximum of 1.4 mmol/L at 10 reps, 2.6 mmol/L at 20 reps and 2.9 mmol/L at 30 reps (Table 3). Perceived exertion increased significantly at higher repetitions, although most parameters hardly differed between 20 and 30 repetitions. There were significant pulmonary effects that were large for RR, ${\dot{V}}_{E}$, ${\dot{V}}_{O_{2}}$ and ${\dot{V}}_{CO_{2}}$. All pulmonary parameters differed significantly between 10 and 20 as well as between 10 and 30 repetitions and revealed an increasing trend with more repetitions, as illustrated in Figure 3.

### 3.3 Mean cardiopulmonary response during ST sessions

Table 4 presents our analysis of the mean values of the ST sessions. Except for TPR, all parameters displayed the lowest values at 10 repetitions compared to 20 and 30 repetitions. The differences between 20 and 30 repetitions are minimal and only significant in HR (129.6 (18.2) vs. 139.8 (10.4); *P* = 0.037) and diastolic blood pressure (115.8 (16.1) vs. 130.3 (16.8); *P* = 0.018) (Table 4). The most noticeable effects are observed in the haemodynamic parameters HR, CO, MAP and TPR. The graphs in Figure 2 also demonstrate a continuous increase in HR, CO and SBP/DBP with higher repetition numbers. TPR was lower at higher numbers of repetitions. There were no significant differences in SV and EDV.

**TABLE 4:** Mean haemodynamic and pulmonary responses during exercise period (*n* = 15, three sets of 10/20/30 repetitions, excluding rest periods)

	**10 repetitions**	**20 repetitions**	**30 repetitions**	**Effect size η^2^_p_**	***P*‐value**
Haemodynamic outcomes					
HR (bpm)	116.1 (10.6)^b, c^	129.6 (18.2)^a, c^	139.8 (10.4)^a, b^	0.65	**0.001**
SV (mL)	85.2 (16.1)^b^	95.3 (20.9)^a^	91.2 (16.9)	0.17	0.092
CO (L/min)	9.84 (1.68)^b, c^	12.42 (3.38)^a^	12.79 (2.51)^a^	0.43	**0.002**
EDV (mL)	163.9 (31.1)	170.0 (26.7)	159.9 (26.4)	—	0.189
SBP* (mmHg)	168.2 (14.0)	176.0 (17.1)	182.2 (21.8)	0.27	**0.033**
DBP* (mmHg)	111.3 (7.1)^c^	115.8 (16.1)^c^	130.3 (16.8)^a, b^	0.45	**0.0004**
MAP* (mmHg)	132.2 (8.5)^c^	140.6 (13.9)	150.7 (18.0)^a^	0.44	**0.001**
RPP*	20067 (3378)^b, c^	23285 (4265)^a, c^	25976 (3772)^a, b^	0.72	**0.001**
TPR* (mmHg min/L)	13.9 (2.7)^b^	12.1 (3.4)^a^	12.3 (3.1)	0.27	**0.017**
SW (J)	1.51 (0.30)^c^	1.80 (0.45)	1.84 (0.43)^a^	—	**0.017**
Pulmonary outcomes					
RR (bpm)	23.2 (5.6)^b, c^	28.1 (8.3)^a^	30.1 (7.1)^a^	0.49	**0.0002**
*V*_T_ (L)	1.35 (0.34)^b, c^	1.59 (0.48)^a^	1.55 (0.41)^a^	—	**0.0006**
${\dot{V}}_{E}$ (L/min)	29.14 (4.36)^b, c^	41.76 (10.51)^a^	44.15 (8.42)^a^	0.67	**0.001**
${\dot{V}}_{O_{2}}$ (mL/min)	1.1 (0.1)^b, c^	1.4 (0.3)^a^	1.4 (0.3)^a^	0.61	**0.001**
${\dot{V}}_{O_{2}}$ (mL/min/kg)	13.4 (1.5)^b, c^	17.2 (3.0)^a^	17.7 (2.9)^a^	0.61	**0.001**
${\dot{V}}_{CO_{2}}$ (mL/min)	1.0 (0.1)^b, c^	1.3 (0.3)^a^	1.4 (0.2)^a^	0.70	**0.001**

*Note*: Values are presented as means and standard deviations. reps, repetitions. *n = 14 (due to technical malfunction of blood pressure monitor). η^2^_p_, partial eta‐squared of the one‐way repeated measures ANOVA. *P*‐values shown in bold indicate statistical significance. ^a^Different from 10 repetitions (*P* < 0.05). ^b^Different from 20 repetitions (*P* < 0.05). ^c^Different from 30 repetitions (*P* < 0.05). Abbreviations: CO, cardiac output; DBP, diastolic blood pressure; EDV, end‐diastolic volume; HR, heart rate; MAP, mean arterial pressure; PPP, peripheral pulse pressure; RPP, rate pressure product; RR, respiratory rate; SBP, systolic blood pressure; SV, stroke volume; SW, stroke work; TPR, total peripheral resistance; ${\dot{V}}_{CO_{2}}$, carbon dioxide output; ${\dot{V}}_{E}$, minute ventilation; ${\dot{V}}_{O_{2}}$, oxygen uptake; *V*_T_, tidal volume.

### 3.4 Cardiopulmonary response in the post‐exercise period (first minute)

In Table 2, our analysis shows the mean values during the first 60 s of the recovery periods after the exercise intervals. We observed significant differences between all training sessions in the HR recovery after exercise (112.8 (12.8) vs. 127.4 (22.0) vs. 141.8 (15.0); *P* = 0.001). As Figure 2a illustrates, HR reached higher values at 30 reps and recovered proportionally slower in the resting periods between exercise intervals. There was an abrupt increase in SV after the exercise intervals (Figure 2b), while blood pressure dropped rapidly after exercise, temporally falling below the initial values (Figure 2d).

We also noted an immediate increase in *V*_T_ (Figure 3b). All pulmonary parameters were significantly higher during the first minute of the resting period at 20 and 30 repetitions compared to 10 repetitions, with no significant differences between 20 and 30 repetitions. Similar results were seen in the lactate values during the recovery phase. Additionally, the participants reported significantly higher ratings of perceived exertion (RPE) in conjunction with rising numbers of repetitions after 60 s of recovery (2.4 (1.2) vs. 3.8 (1.5) vs. 5.4 (1.4); *P* = 0.001).

Table 5 presents the difference (delta values) between the mean values before the exercise (120 s before exercising 10/20/30 reps) and peak values achieved at 10, 20 or 30 repetitions (last 30 s). These data were compared using ANOVA or Friedman's test after 10 repetitions (10/20/30 repetitions) and Student's paired *t* test after 20 repetitions (20/30 repetitions).

**TABLE 5:** Delta values of pre‐interval values to peak values at 10/20/30 reps (*n* = 15).

	**δ value of pre‐interval to peak at 10 reps**	**Effect size η^2^_p_ δ at 10 reps**	***P*‐value** **δ at 10 reps**	**δ value of pre‐interval to peak at 20 reps**	**Effect size η^2^_p_ δ at 20 reps**	***P*‐value** **δ at 20 reps**	**δ value of** **pre‐interval to peak at 30 reps**
HR (bpm)							
10 reps 20 reps 30 reps	31.1 (9.4) 34.2 (9.8) 30.8 (9.5)	0.16	0.096	46.6 (14.0) 44.9 (10.5)	0.03	0.514	52.7 (12.1)
SV (mL)							
10 reps 20 reps 30 reps	8.22 (8.90) 8.64 (6.42) 5.18 (7.54)	0.10	0.248	10.4 (7.0) 8.55 (5.9)	0.06	0.343	11.67 (6.52)
CO (L/min)							
10 reps 20 reps 30 reps	3.60 (1.14) 4.11 (1.76) 3.33 (1.30)	—	0.165	5.57 (2.22) 5.10 (1.66)	0.06	0.369	6.32 (2.10)
SBP (mmHg)*							
10 reps 20 reps 30 reps	31.3 (14.9) 29.6 (13.8) 28.0 (14.2)	0.03	0.696	26.7 (23.6) 36.6 (20.2)	0.28	**0.042**	29.4 (23.7)
DBP (mmHg)*							
10 reps 20 reps 30 reps	18.5 (12.4) 19.9 (15.2) 20.9 (11.2)	0.02	0.771	19.8 (20.5) 31.4 (17.0)	0.28	**0.043**	28.3 (20.4)
MAP (mmHg)*							
10 reps 20 reps 30 reps	23.7 (13.3) 24.6 (13.2) 23.3 (11.6)	0.01	0.913	24.3 (20.5) 34.1 (17.0)	0.26	0.054	30.2 (20.2)
RPP*							
10 reps 20 reps 30 reps	7977 (2234) 8541 (1880) 8110 (2619)	0.05	0.503	10295 (3657) 11726 (3449)	0.29	**0.039**	12314 (3903)
TPR (mmHg min/L)*							
10 reps 20 reps 30 reps	−3.27 (1.75) −2.79 (2.06) −2.24 (1.37)	0.13	0.159	−4.06 (2.62) −3.04 (1.49)	0.12	0.202	−4.25 (1.64)

*Note*: Values presented as the means and standard deviation. reps, repetitions. *n = 14 (due to technical malfunction of blood pressure monitor). η^2^_p_, partial eta‐squared of the one‐way repeated measures ANOVA and Student's *t*‐test. *P*‐values shown in bold indicate statistical significance. Abbreviations: CO, cardiac output; DBP, diastolic blood pressure; HR, heart rate; MAP, mean arterial pressure; RPP, rate pressure product; SBP, systolic blood pressure; SV, stroke volume; TPR, total peripheral resistance.

## 4 DISCUSSION

Our participants’ haemodynamic and vascular responses (HR, CO, DBP) to different repetitions in ST indicated significantly increased amplitude with greater repetitions. This association is not apparent for peak SV, SBP and EDV (SBP differs significantly in mean values) and is the inverse for the TPR. However, there were already differences in HR and DBP at baseline, indicating a pre‐start reaction (Decety et al., 1991). The pulmonary and metabolic values show an even more pronounced increase with more repetitions, reflecting prolonged muscular effort. In particular, inadequate HR recovery due to the cumulative repetition‐dependent workload after 20 and especially 30 repetitions led to significantly higher HR and DBP values. There were no differences in end‐diastolic filling. The increased stroke work when performing the 30‐repetition protocol is a response to exercise and post‐exercise time, with an associated rise in DBP.

The ‘strength–endurance or repetition continuum’ suggests specific training adaptations in high load/few repetitions versus low load/high repetitions. Training with a high load (few repetitions) is known to increase 1‐RM strength, while a low load (many repetitions) induces positive effects on aerobic capacity (Carvalho et al., 2022; Schoenfeld et al., 2015). Muscle hypertrophy can be achieved across various loading ranges (Carvalho et al., 2022; Schoenfeld et al., 2015). Low load/high repetition ST significantly increases aerobic performance and time to exhaustion without increasing ${\dot{V}}_{O_{2}\max}$, compared to high load/low repetitions (Campos et al., 2002). These authors assume that the results they observed are attributable to the economisation of movement patterns (Campos et al., 2002). In terms of acute haemodynamic effects, very high strength intensities or isometric loads have been reported to cause elevated blood pressure which is due to the increasing TPR and HR (Haslam et al., 1988; MacDougall et al., 1992; Taylor et al., 2017). Blood pressure responses to low‐load training appear to be less pronounced due to the absence of the Valsalva manoeuvre (Niewiadomski et al., 2012). However, there is initial evidence suggesting that low‐load training with many repetitions may lead to more robust cardiovascular responses compared to high‐load training (Gjovaag et al., 2016; Lamotte et al., 2010, 2005). In conjunction with our published study (Lässing et al., 2023), this study aimed to continuously measure the acute haemodynamic and blood pressure responses separately for each training factor, specifically repetition and intensity.

### 4.1 Cardiovascular response in baseline, exercise and post‐exercise period

The *baseline* cardiovascular parameters SV, CO, SBP, TPR, RPP and SW revealed no differences between training sessions. Only HR and DBP were higher before the training with 30 repetitions. These findings stand in contrast to those of other studies (Gjovaag et al., 2016; Lamotte et al., 2010; Machado et al., 2020). Nevertheless, evidence suggests that the central feedforward mechanism, resulting from the anticipated workload (expected higher number of repetitions), can raise the HR dependent on the subsequent exercise intensity (Decety et al., 1991; Miyamoto et al., 2022).

A progressive increase in CO and HR from set to set during the *exercise period* is apparent in Figure 2 and Table 2. Lamotte et al. (2005), and Gjovaag et al. (2016) found similar increased CO and HR responses with higher repetitions despite less intensity (same workload) in contrast to lower repetitions and higher intensity (Gjovaag et al., 2016; Lamotte et al., 2005). These enhanced cardiac responses are also caused by isolated higher intensity (different workload) (Lässing et al., 2023) and with increasing repetitions at the same intensity (current results).

The peak and mean values of the SV response during the condition with 20 and 30 repetitions showed significantly higher values than with 10 repetitions (Table 2). We assume that the higher SV is caused by the significantly higher HRs and is associated with the Bowditch effect, inducing stronger cardiac contractility (Erbel et al., 1979; Higginbotham et al., 1986; Usman et al., 2024). On the other hand, Gjovaag et al. (2016) proposed that a higher EDV could lead to a higher SV as per the Frank–Starling mechanism. However, our comparison of training sessions during exercise showed no changes in EDV (Table 2).

The regulation of blood pressure that depends on repetition numbers results in the SBP's responses being more differentiated than the DBP's responses (DBP rose successively with repetitions; Table 2). Sousa et al. (2014) showed that blood pressure rises somewhat linearly during the squat at 50% of 1‐RM in the leg press, reaching the highest value at the end of the exercise. The authors assume a rise in TPR and HR‐induced CO, which raises blood pressure with successive repetitions or intensities (de Sousa et al., 2014). These results indicate that the haemodynamic and cardiovascular responses to higher repetition and intensity protocols are subject to roughly the same regulation (de Sousa et al., 2014; Lamotte et al., 2005).

**FIGURE 4:**
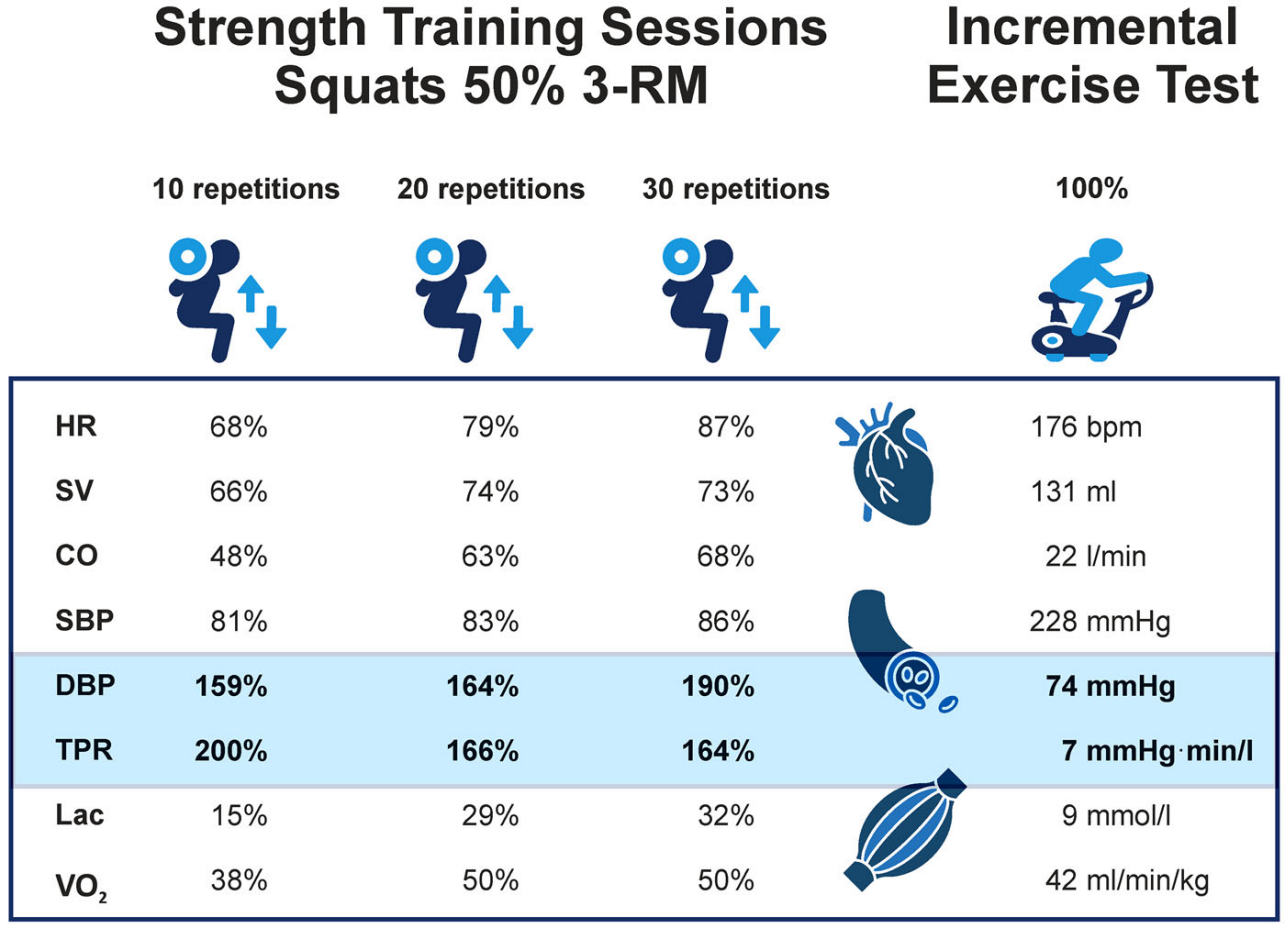
Diagram illustrating the acute cardiac, vascular and metabolic responses to training sessions. These data correspond to the 100% values in an incremental exercise test on an ergometer. Overall, as the number of repetitions increased, there was a progressively stronger cardiac response (HR, SV, CO), but it was lower in comparison to IET. TPR during strength training is two‐fold higher than TPR in the IET, but it decreases with more repetitions due to metabolic changes. As a result of the increased TPR, the DBP also rose compared to the IET. Unlike TPR, however, the DBP increased with more repetitions, which may be attributed to the rise in CO with more repetitions.

Our TPR results indicate a decrease during and after exercise, with a more substantial effect observed with more repetitions (Table 2). However, the TPR is two‐fold higher than the IET (Figure 4). The isolated TPR effect during exercise on raising blood pressure has been poorly studied. Most studies have investigated isometric or high‐intensity exercise using the Valsalva manoeuvre (Lentini et al., 1993; MacDougall et al., 1992, 1985; Niewiadomski et al., 2012). We observed a marked increase in DBP during 30 repetitions compared to 20 repetitions, while CO and TPR remained similar (Table 2). We, therefore, assume a cumulative effect with increasing repetitions, as supported by the successive rise in HR and CO during the set resting intervals (see Figures 2 and 3). Despite the similarity in SV values across the training sessions, especially at 20 and 30 repetitions, this seems to be attributable to the increase in blood pressure and the compensatory mechanism of a higher HR (Hietanen, 1984). Certain studies suggest that dynamic and isometric force stimuli cause an early return of pulse wave reflections, leading to an increase in pulsatile afterload (Stock et al., 2020; Wilkinson et al., 2000). When diastolic durations are shortened (HR), the duration of the diastolic outflow rate and shortened absolute ejection phase result in a shift in the pulse wave during the diastolic period, leading to higher DBP (Wilkinson et al., 2000).

HR values in the three training sessions varied significantly during the *post‐exercise periods* (highest with 30 repetitions). Additionally, there was a notable increase in SV during the first post‐exercise minute. CO in the recovery phase remained largely unchanged in the 10 repetitions condition but was markedly higher during training sessions requiring 20 and 30 repetitions, similar to the exercise period. DBP revealed differences between the 30 repetitions and the two other conditions. TPR was lowest after 30 repetitions (Table 2). These findings suggest a causal relationship between higher post‐exercise HRs, consistent with peak exercise HRs, and the different post‐exercise regulation of blood pressure.

Rezk et al. (2006) found that the HR rose during the post‐exercise period, especially at high intensities/few repetitions. They suggest that TPR increases during high load/low repetition conditions to compensate for falling SBP (Rezk et al., 2006). In contrast, our data failed to reveal an HR increase in the post‐exercise period. Similar SBP values between training sessions were due to the lower TPR, especially at high repetition numbers. This resulted in significantly reduced DBP between the exercise to post‐exercise periods with high repetition numbers, similar to evidence from isometric strength exercises (Edwards et al., 2022).

### 4.2 Pulmonary and metabolic response in baseline, exercise and post‐exercise periods

Our *baseline* pulmonary parameters (*V*_T_, ${\dot{V}}_{E}$, ${\dot{V}}_{O_{2}}$, ${\dot{V}}_{CO_{2}}$) differed before the exercise period, even though we had randomised the training sessions. This could imply an increase in central forward regulation depending on anticipated exercise and associated demands (Miyamoto et al., 2022).

During the *exercise periods*, our breathing data (RR, *V*_T_, ${\dot{V}}_{E}$) were significantly higher at 20 and 30 repetitions as opposed to 10 repetitions. However, the results from the training sessions at 20 and 30 repetitions showed no difference. This could be due to breath holding or employing the Valsalva manoeuvre during the higher repetitions sets and the cumulative effort involved in performing numerous repetitions. The higher BP and *V*_T_ withdrawal behaviour over longer repetition periods at 20 and 30 repetitions may also suggest breath holding during the sets.

The levels of ${\dot{V}}_{O_{2}}$ and ${\dot{V}}_{CO_{2}}$ were also higher during 20 and 30 repetitions than during 10 repetitions. These differences in ventilation appear to be influenced by higher sympathetic activation and potentially greater resetting of the baroreflex during the high repetition condition. This may be mediated by pressoreflex responses involving afferent sensory neurons in group IV (metabolic) and group III (mechanical) (Fisher et al., 2008; Laginestra et al., 2023).

The metabolic (blood lactate concentration) and subjective (RPE) efforts were highest in the training sessions requiring 20 and 30 repetitions, similar to pulmonary parameters. Confirming this, Gjovaag et al. (2016) also reported higher lactate concentrations and RPE values after exercise when doing 20 repetitions at 40% of MVC versus four repetitions at 90% of MVC.

Acute differences in repetitions may trigger long‐term increases in pulmonary and metabolic parameters, but the extent of this effect is uncertain. In agreement with Schoenfeld et al. (2015), we assume that the higher pulmonary and metabolic demands during training sessions with more repetitions can lead to long‐term cellular phenotypic adaptations.

In summary, the changes observed here in the number of repetitions at the same intensity indicate notable differences in pulmonary and metabolic response reactions. These discernible differences do not correlate with increased intensity at the same number of repetitions (Lässing et al., 2023). Higher repetition numbers seem to align with the metabolic demands of high intensities (75% 1‐RM), with the oxygen uptake being more significantly influenced by the repetition numbers during exercise.

Relying on the peak values at the maximal load level in the IET, Figure 4 schematically depicts the cardiovascular regulation related to the number of repetitions in ST sessions.

In addition to the maximum values of the IET in Figure 4, Table 1 presents the cardiopulmonary response at the ventilatory thresholds. The intensity at these thresholds is often used to make training recommendations for both extensive and intensive endurance training. When performing ST with 30 repetitions, we observed similar mean responses in HR, CO and blood lactic acid concentration (Table 4) as in the range between the ventilatory thresholds (Table 1). Mean SBP and pulmonary parameters (${\dot{V}}_{E}$, ${\dot{V}}_{O_{2}}$) reached the level of VT1 during the IET (Table 4). In contrast, DBP and TPR displayed significantly higher deflections during ST than dynamic endurance exercise. Based on these data, it appears that ST with exhaustive repetitions at 50% of the 3‐RM may potentially elicit similar cardiac, but not pulmonary, responses as endurance training. However, it is important to note the potential impact of peripheral vascular compression resulting from the high strength requirements in ST. Compared to endurance training, this compression may lead to metabolic and pulmonary differences.

### 4.3 Study limitations

Our study has a small sample size and included only male participants engaging in recreational activities. Therefore, the results of our study are limited in their interpretability and generalisability, and they apply only to a healthy male population. Studies have shown differences in blood pressure regulation between sexes at rest and during dynamic stress (Bassareo & Crisafulli, 2020; Bauer et al., 2021; Briant et al., 2016; Williams et al., 2017). It remains essential to study a female collective to identify gender‐specific regulatory differences. Nonetheless, this trial is the largest randomised crossover study to date investigating the acute haemodynamic responses outside the ‘strength–endurance continuum’ by increasing repetitions without changing intensity. It is worth noting that cardiac parameters obtained through impedance cardiography may be overestimated when using absolute values (Siebenmann et al., 2015). However, when comparing intra‐individual differences, changes in these parameters were more relevant than the absolute values. Other research groups have used thoracic impedance cardiography to identify intra‐individual changes in SV and CO (Charloux et al., 2000; Richard et al., 2001). Impedance cardiography measurements of SV and CO demonstrate good agreement compared to magnetic resonance imaging and direct Fick or pulmonary thermodilution methods (Siebenmann et al., 2015).

### 4.4 Conclusions

The ACSM Guidelines for Exercise Testing and Prescription recommend a higher number of repetitions and an intensity of 40–50% of 1‐RM for muscular fitness improvements. Our findings from intensity‐matched ST demonstrate that performing more than 20 repetitions, leading to muscular exhaustion also elicits a significant cardiopulmonary response. This includes a significant increase in diastolic blood pressure (DBP) and an accelerated HR, similar to the effects of isometric exercise. This study supports evidence demonstrating a more robust cardiovascular response with low load and high repetition protocols compared to high load/few repetition protocols (Gjovaag et al., 2016; Lamotte et al., 2005). Considering these results, we believe the repetition‐dependent haemodynamic response could contribute to improving ST recommendations in recreational and competitive sports.

## AUTHOR CONTRIBUTIONS

Johannes Lässing and Roberto Falz conceived and designed this research. Sonja Hummelmann and Maxi Kramer conducted experiments. Johannes Lässing, Sonja Hummelmann, Sven Fikenzer and Ulrich Laufs analysed and interpreted the data. Johannes Lässing, Sonja Hummelmann, Sven Fikenzer, Ulrich Laufs and Roberto Falz drafted the manuscript. Johannes Lässing, Sonja Hummelmann, Maxi Kramer, Sven Fikenzer, Ulrich Laufs and Roberto Falz. have revised the manuscript. All authors have read and approved the final version of this manuscript and agree to be accountable for all aspects of the work in ensuring that questions related to the accuracy or integrity of any part of the work are appropriately investigated and resolved. All persons designated as authors qualify for authorship, and all those who qualify for authorship are listed.

## CONFLICT OF INTEREST

The authors have no competing interests to declare and have no relevant financial or non‐financial interests to disclose.

## FUNDING INFORMATION

No funding was received for this work.

## Supporting information



Raw data hemodynamic parameters

Raw data pulmonary parameters

## Data Availability

The original contributions presented in the study are included in the article's supplementary material; further inquiries can be directed to the corresponding author.
